# Reliability of Immersive Virtual Reality for Pre-Procedural Planning for TAVI: A CT-Based Validation

**DOI:** 10.3390/jcdd12120481

**Published:** 2025-12-08

**Authors:** Nicole Carabetta, Giuseppe Panuccio, Salvatore Giordano, Sabato Sorrentino, Giuseppe Antonio Mazza, Jolanda Sabatino, Giovanni Canino, Isabella Leo, Nadia Salerno, Antonio Strangio, Maria Petullà, Daniele Torella, Salvatore De Rosa

**Affiliations:** 1 Department of Medical and Surgical Sciences, Magna Graecia University, 88100 Catanzaro, Italy; nicole.carabetta95@gmail.com (N.C.); giuseppeantoniom@gmail.com (G.A.M.); 2 ”R. Dulbecco” University Hospital, 88100 Catanzaro, Italy; panuccio@unicz.it (G.P.); sabatino@unicz.it (J.S.); canino@unicz.it (G.C.);; 3 Cardiovascular Research Center, Magna Graecia University, 88100 Catanzaro, Italy; 4 Department of Experimental and Clinical Medicine, Magna Grecia University, 88100 Catanzaro, Italy; 5 Department of Cardiology, Angiology and Intensive Care Medicine, Deutsches Herzzentrum der Charité, 12200 Berlin, Germany

**Keywords:** computed tomography, structural heart disease, virtual simulation, transcatheter aortic valve replacement

## Abstract

**Background.** Accurate anatomical assessment is essential for pre-procedural planning in structural heart disease. Advanced 3D imaging could offer improved visualization for more accurate reconstruction. We assessed the performance of a novel immersive 3D virtual reality (VEA) for the pre-procedural planning of transcatheter aortic valve implantation (TAVI) candidates. **Methods.** Measurement of cardiac-gated contrast-enhanced computed tomography (CT) scans was performed with the novel VEA and established tools: 3Mensio and Horos. **Results.** 50 consecutive patients were included. Annular and LVOT measurements obtained with VEA were strongly correlated with those derived from standard CT analysis. The intraclass correlation coefficient (ICC) confirmed excellent consistency for annular measurements (ICC = 0.93), while the concordance correlation coefficient indicated very good overall agreement (CCC = 0.83, 95% CI 0.73–0.90). Similarly, LVOT measurements obtained with VEA showed strong correlation with CT values, with good consistency (ICC = 0.90) and good overall agreement (CCC = 0.77, 95% CI 0.64–0.86). VEA-based planning improved prosthesis size selection accuracy, achieving higher concordance with implanted valves and a significant net reclassification gain over conventional CT. **Conclusions.** Given the increasing use of advanced 3D cardiac imaging technologies, understanding their diagnostic accuracy to guide pre-procedural planning of TAVI is paramount. In our study, VEA provided reliable assessment of aortic root anatomy for TAVI planning. This novel 3D software provides accurate, patient-specific reconstructions of the aortic root and surrounding structures that may optimize valve sizing, improve procedural safety and enhance procedural outcomes. This provides a rationale for future studies to assess the procedural benefit derived from a three-dimensional assessment of the aortic valve geometry.

## 1. Introduction

The management of aortic valve stenosis has undergone a paradigm shift over the last two decades with the rapid adoption of transcatheter aortic valve implantation (TAVI). TAVI has become a cornerstone of modern structural heart interventions, offering a less invasive alternative to surgical aortic valve replacement (SAVR), with comparable or superior outcomes in selected populations, fostering the extension of indications to TAVI [1].

Cardiac imaging represents a fundamental tool to plan and optimize percutaneous interventions [2,3]. The continuously expanding target patient population demands meticulous preoperative planning to warrant the optimal success of TAVI [4,5]. The planning relies heavily on multidetector computed tomography (CT) imaging to evaluate aortic root anatomy, access vessels, and potential procedural risks, in addition to advanced echocardiography to assess the clinical indication and characterize both valvular and cardiac function [6,7,8,9].

In this context, emerging technologies have begun to reshape how preoperative data are interpreted and utilized [10,11]. Among these, virtual reality (VR) has gained increasing attention for its potential to enhance anatomical understanding and spatial awareness. VR allows immersive, three-dimensional visualization of CT data, enabling clinicians to interact with patient-specific anatomy in a fully navigable environment. This may be particularly valuable in complex cases, such as those with heavily calcified valves, borderline annular dimensions, or challenging anatomies [12,13].

Recent studies have suggested that VR can facilitate improved communication among multidisciplinary heart teams, aid in pre-procedural simulation, and potentially reduce intraoperative uncertainty. As such, VR represents a promising tool in the evolving landscape of preoperative planning, offering a novel approach to individualized procedural strategy [14].

Ruyra et al. found that in nearly half of the 11 TAVI cases assessed, new insights from VR led to a redesign of the procedural plan, consistently increasing the confidence of the Heart Team [15]. Similarly, in a pilot study of ascending aortic surgery, 33% of preoperative decisions were adjusted after VR evaluation, and 80% of surgeons felt better prepared compared to conventional imaging [16].

In this article, we describe the integration of immersive VR into the TAVI planning workflow, focusing on its application in visualizing preoperative CT scans. In fact, while previous works have explored the qualitative value of VR in enhancing anatomical understanding and team communication, data on quantitative validation of VR-derived anatomical measurements for TAVI planning are scarce. We therefore aimed to assess VR not only as a tool to enhance the operator’s understanding of the three-dimensional anatomy of the aortic root but also to explore its potential in providing direct anatomical measurements relevant for TAVI planning. In addition, we meant to test the concordance between the prosthesis virtually selected based on VEA measurements and the one that was actually selected in study patients. We also discuss the technical aspects, clinical utility, and future implications of VR in structural heart disease interventions, with an emphasis on its role in enhancing the precision and safety of transcatheter aortic valve replacement.

## 2. Methods

### 2.1. Study Design and Population

We retrospectively included 50 consecutive patients with severe aortic stenosis who were assessed for eligibility for transcatheter aortic valve implantation (TAVI) at Magna Graecia University, Catanzaro, Italy. All patients underwent multislice computed tomography (CT) as part of the standard pre-procedural evaluation. Selection criteria for inclusion were clinical indication for TAVI and minimal quality requirements for CT imaging, such as adequate contrast enhancement of the aortic root; absence of significant motion artifacts; and sufficient spatial resolution to allow accurate measurements of the annulus, left ventricular outflow tract (LVOT), sinotubular junction, ascending aorta, and coronary heights. In addition to imaging data, baseline clinical and procedural characteristics were also collected, including balloon pre-dilatation and post-dilatation. Peri-procedural and in-hospital complications were systematically recorded. Post-procedural transthoracic echocardiography was performed in all patients to evaluate valve function and detect possible complications such as paravalvular leak or prosthesis dysfunction. Valve sizing during the TAVI procedures was based on pre-procedural CT measurements performed by the interventional team according to standard clinical practice, considering annular dimensions, calcium distribution and coronary ostia height. For the retrospective analysis, both the conventional CT and the VEA measurements were repeated by different operators, who were not involved in the clinical procedures and were blinded to the intra-procedural decisions. This approach ensured consistency in the retrospective evaluation, although minor discrepancies between the original and repeated measurements may occur. The implanted valve was maintained as the reference device, while only the virtual sizing was adjusted to assess differences between CT-based and VEA-based planning.

### 2.2. Multislice Computed Tomography and Software Measurements

CT acquisitions were performed in different laboratories using multislice CT scanners from various companies (GE, Healthcare, Chicago Illinois, USA; Siemens Healthineers, Erlangen, Germany). Acquisition protocols followed local institutional standards for pre-TAVI imaging and were consistent with current international guidelines [17]. A contrast-enhanced, ECG-gated CT of the thorax was performed to evaluate the aortic root and LVOT, followed by a non-ECG-synchronized helical CT of the abdomen and pelvis. Images were acquired during a single breath-hold after intravenous injection of iodinated contrast medium (contrast volume 50–100 mL) with a flow rate of 4–6 mL/s. ECG gating was retrospective, with image reconstruction performed from 20% to 80% of the cardiac cycle. Datasets for standard CT assessment were analyzed using two different software platforms: Horos (version 3.3.6) and 3Mensio Structural Heart (version 10.7, Pie Medical Imaging, Maastricht, The Netherlands). In both cases, measurements of the LVOT area, aortic annulus area, sinotubular junction, ascending aorta, and coronary ostial heights were obtained according to current guidelines. Multiplanar reconstruction was used to ensure accurate alignment, and all measurements were performed at 75% of the cardiac cycle.

### 2.3. Visualization of the Data in Virtual Reality

The same datasets were imported into VEA (*Virtual simulations* sp.), a dedicated three-dimensional (3D) visualization tool based on immersive virtual reality. Measurements of LVOT and annulus area were performed at 75% of the cardiac cycle, ensuring direct comparability with conventional analysis. The system includes a virtual reality headset and a handheld controller that allow the operator to interact with the 3D model in real time by slicing multiple planes, removing overlying structures, changing the view angle, and performing precise measurements on the reconstructed anatomy.

### 2.4. Assessment of Reproducibility

Experienced imaging specialists performed all measurements to analyze the interobserver variability. To analyze the intraobserver reliability, 1 physician repeated the measurements of 10 patients 3 months later. Inter- and intraobserver variability for VR and conventional CT measurements were evaluated using the intraclass correlation coefficient (ICC).

### 2.5. Statistical Analysis

Analyses were performed using IBM SPSS Statistics version V.30.0 for Windows (IBM Corp., Armonk, NY, USA). Shapiro–Wilk tests were used to assess the normal distributions among the analyzed data. Continuous variables were shown as mean ± standard deviation or as median and interquartile range in cases of non-normal distribution. Frequency and percentage were used to summarize categorical variables. Data with normally distributed samples were compared using Student’s two-sample paired *t*-test, and data with non-normally distributed samples were compared using the Wilcoxon test. To assess the agreement between CT and VEA measurements, we employed complementary statistical and graphical approaches. A scatter plot with the line of identity was generated to visually assess the correlation between the two methods. The strength of correlation and precision was quantified through Pearson’s correlation coefficient and the intraclass correlation coefficient (ICC), calculated for both single and average measures. Agreement was further evaluated using the concordance correlation coefficient (CCC), which accounts for both precision and accuracy by combining Pearson’s ρ with a bias correction factor. In addition, a Bland–Altman plot was constructed to estimate the mean bias and 95% limits of agreement, thereby identifying potential systematic differences between methods. To better visualize the distribution of measurement discrepancies, a histogram of differences was generated, illustrating whether values clustered around zero or displayed systematic spread. Finally, dot plots were employed to directly compare individual paired measurements from CT and VEA, providing an intuitive visualization of the overlap between methods. Agreement between the planned prosthesis size and the actually implanted valve was assessed using multiple complementary approaches. First, the mean absolute error (MAE) was calculated for each method (CT- and VEA-based planning, separately for two different operators) as a continuous measure of deviation from the implanted size. In addition, exact concordance rates were computed as the proportion of cases in which the planned prosthesis size was identical to the implanted valve. To further quantify categorical agreement, Cohen’s kappa coefficients were calculated between planned and implanted valve sizes. Bland–Altman analyses were also performed to evaluate bias and limits of agreement between planned and implanted sizes. As multiple software programs were used to analyze CT scans, inter-software variability assessment was performed in a subgroup of 10 randomly selected patients using the ICC. For the reclassification analysis, each case was classified as “better” if the VEA-based planning reduced the absolute deviation from the implanted valve size compared with the CT-based planning (or achieved exact concordance when CT did not), “worse” if the deviation increased (or concordance was lost), or “no change” if accuracy was unchanged. The proportions of cases falling into each category were reported, and a Net Reclassification Improvement (NRI) index was calculated as (*N*_better_ − *N*_worse_)/*N*_total_. Exact binomial tests were applied to evaluate the statistical significance of the observed reclassification direction, and 95% confidence intervals for the NRI were estimated using Wilson’s method. The implanted valve was used as a reference comparator. Statistical significance was assumed if the *p*-value was <0.05.

## 3. Results

### 3.1. Study Population

This study included 50 consecutive patients with severe aortic stenosis referred to our center to assess eligibility for transcatheter aortic valve implantation (TAVI). Of these, 7 patients did not ultimately undergo TAVI due to unsuitable vascular access (*n* = 2), patient refusal (*n* = 3), or referral to cardiac surgery *(n* = 2). The median age was 80.5 years (IQR = 77–85.8), and patients were equally distributed between male and female sexes. Cardiovascular risk factors such as hypertension (91%), dyslipidemia (94%), and diabetes (42.5%) were highly prevalent. A history of chronic kidney disease was observed in 12 patients (25.5%), prior myocardial infarction in 6 patients (12.7%), and previous percutaneous coronary intervention (PCI) in 8 patients (17%), while prior coronary artery bypass grafting (CABG) and valve surgery were less common, with 2 patients (4%) and 1 patient (2.1%), respectively. Atrial fibrillation was documented in 11 patients (23.4%), and 3 patients (6.3%) had a prior pacemaker implantation. Table 1 reports a detailed description of the study population.

CT from all study patients was assessed both via immersive virtual reality using VEA and with traditional CT measurement software: one half *(n* = 26) with Horos and the other half (*n* = 24) with 3Mensio Structural Heart.

Baseline echocardiographic characteristics are summarized in Table 2. The median left ventricular ejection fraction (LVEF) was 57% (IQR 53.5–59.75), and the median left atrial volume index (LAVi) was 47 mL/m^2^ (IQR 38.25–53.5). The median peak aortic velocity was 4.19 m/s (IQR 3.9–4.46), with a median mean transaortic gradient of 42.34 mmHg (IQR 38–51.25). The median aortic valve area, calculated using the continuity equation, was 0.7 cm^2^ (IQR 0.6–0.8). The estimated systolic pulmonary artery pressure (sPAP) was 40 mmHg (IQR 35–45).

CT-derived anatomical measurements are reported in Table 3. The median sinotubular junction diameter was 27.5 mm (IQR 25.5–29.55), while the median ascending aorta diameter was 33.6 mm (IQR 29.5–35.8). The median left coronary height was 13.1 mm (IQR 11.5–14.7), and the right coronary height was 13.7 mm (IQR 10.65–15.35).

**Figure 1:**
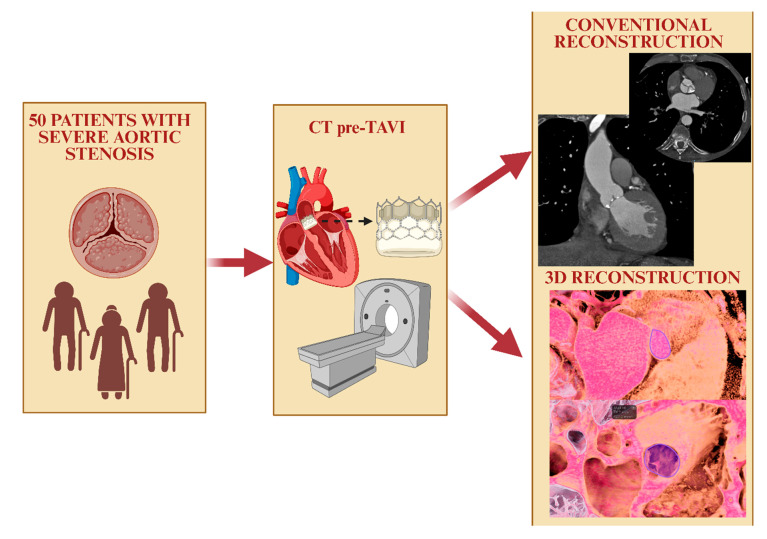
Comparison of conventional and 3D virtual reality reconstruction from pre-TAVI CT in severe aortic stenosis.

**Figure 2:**
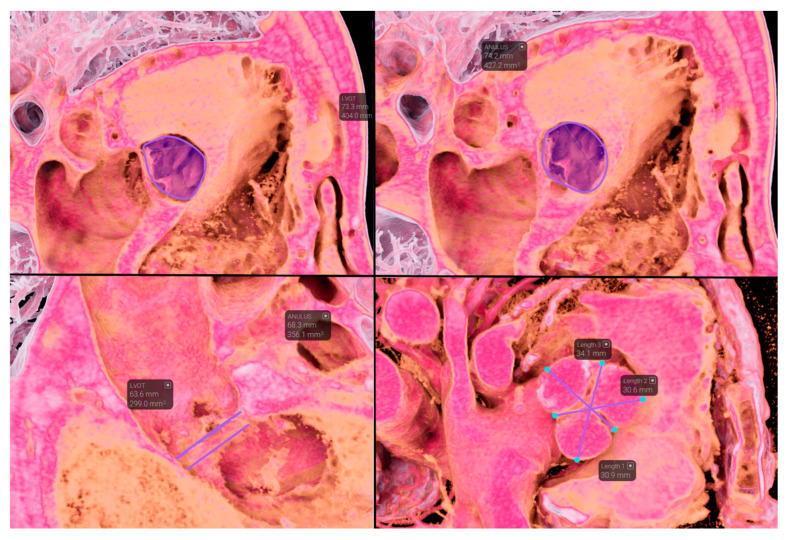
Example of aortic measurements obtained through immersive virtual reality.

Annular and LVOT measurements were obtained with both the standard method and VEA. (Figure 1 and Figure 2). The median annulus area was 428 mm^2^ (IQR 364–464) with the standard method and 398 mm^2^ (IQR 345–444) with VEA. The median LVOT area was 383 mm^2^ (IQR 335–437) with the standard method and 356 mm^2^ (IQR 304–403) with VEA, confirming the interchangeability of the two systems.

**Figure 3:**
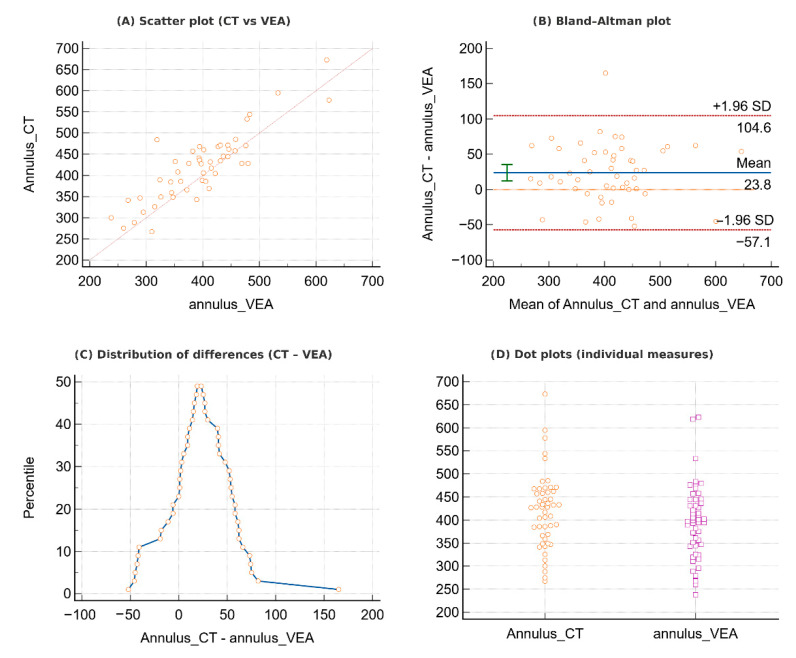
Comparison between CT and immersive virtual reality (VEA) annular measurements. (**A**) Scatter plot showing the correlation between CT and VEA. (**B**) Bland–Altman plot demonstrating mean bias and 95% limits of agreement. (**C**) Distribution of differences (CT–VEA), with most values clustering around zero. (**D**) Dot plots of individual annular measurements obtained with CT and VEA, highlighting their close comparability.

**Figure 4:**
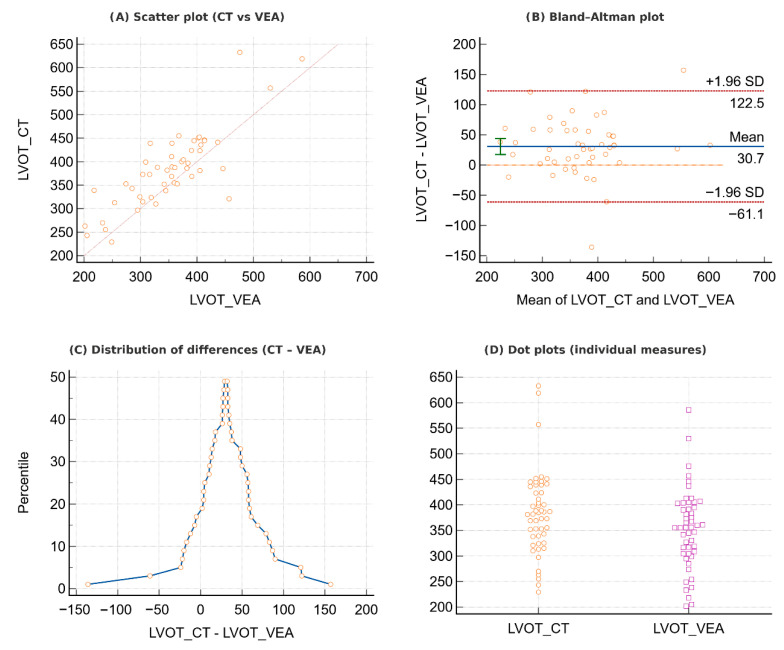
Comparison between CT and immersive virtual reality (VEA) LVOT measurements. (**A**) Scatter plot illustrating correlation between CT and VEA. (**B**) Bland–Altman plot showing mean bias and 95% limits of agreement. (**C**) Distribution of differences (CT–VEA), with clustering around the mean. (**D**) Dot plots of individual CT and VEA values, demonstrating overall comparability between the two methods.

Annular measurements obtained with VEA were strongly correlated with those derived from standard CT measurements (Figure 3A). The intraclass correlation coefficient confirmed excellent consistency (ICC = 0.87 for single measures, 0.93 for average measures), while the concordance correlation coefficient indicated very good overall agreement (CCC = 0.83, 95% CI 0.73–0.90). Bland–Altman analysis (Figure 3B) revealed a modest mean bias (23.8, *p* = 0.0002), yet the distribution of differences (Figure 3C) showed that most values clustered around zero. Direct visualization of CT and VEA measurements in dot plots (Figure 3D) shows close alignment between the two techniques. Similarly, LVOT measurements obtained with VEA were strongly correlated with those from CT (Figure 4A). The intraclass correlation coefficient indicated very good consistency (ICC = 0.83 for single measures, 0.90 for average measures), and the concordance correlation coefficient showed good overall agreement (CCC = 0.77, 95% CI 0.64–0.86). Bland–Altman analysis (Figure 4B) demonstrated a mean bias of 30.7 (*p* < 0.0001), suggesting the presence of systematic differences. Nevertheless, the distribution of differences (Figure 4C) showed that most values clustered around the mean. Finally, direct visualization of individual measurements (Figure 4D) highlighted substantial overlap between the two techniques.

In addition, a comparison of measurements obtained from Horos and 3Mensio was performed in a subset of patients. The analysis demonstrated a high level of agreement between the two platforms (ICC = 0.99, 95% CI 0.96–1.00 for single measures; ICC = 0.995, 95% CI 0.98 for average measures). Overall, among the 50 patients analyzed, 43 patients underwent TAVI. Evolut FX valves (Medtronic, Minneapolis, MN, USA) were the most frequently implanted (18 patients, 41.8%). Navitor valves (Abbott Structural Heart, Chicago, IL, USA) were implanted in 6 patients (13.9%), and Sapien 3 Ultra Valves (Edwards Lifesciences, Irvine, CA, USA) in 10 patients (23.2%). The remaining cases (9 patients) received Evolut FX+ (*n* = 1), PRO (*n* = 1), PRO+ (*n* = 4), R (*n* = 2), or Sapien Ultra + (RESILIA) devices (*n* = 1). Balloon pre-dilation was performed in 10 patients (23%), and post-dilation in 6 patients (13.9%). Following TAVI, 20.1% required new pacemaker implantation, and 13.9% underwent PCI during the procedure. However, among the nine patients who received pacemakers, three already had a pre-existing indication before the TAVI procedure, and implantation was therefore performed after TAVI. When these cases were excluded, the actual rate of new pacemaker implantation was 13%. On the other hand, in patients receiving concomitant PCI, significant coronary stenoses were documented either angiographically or on pre-procedural CT, and revascularization was performed according to current guidelines recommendations. Moreover, 9 patients (29.1%) had mild–moderate regurgitation. Immediately after the procedure, the median mean transaortic gradient was 8.3 mmHg (IQR 6–11.25) (Table 4).

### 3.2. Comparison of Standard and Virtual Reality Pre-Procedural CT Images Assessment

We compared the valve sizes selected during preoperative planning, using both conventional CT-based assessment and the novel VEA, against the actual implanted valve size. When tested by operators expert in structural heart interventions, the mean absolute error (MAE) relative to the implanted valve size was 1.16 mm for CT-based planning and 0.74 mm for VEA-based planning (*p* = 0.014). Exact concordance (identical match with the implanted prosthesis) was achieved in 65.1% of cases for CT-based planning and 79.1% for VEA-based planning (*p* = 0.048). These findings indicate that the VEA-based planning achieved both the lowest error and the highest exact agreement with the implanted valve size. This suggests that the use of VEA may provide a more accurate prosthesis size selection compared with conventional CT-based planning. Cohen’s kappa values showed moderate agreement for CT-based planning (κ = 0.51) and substantial agreement for VEA (κ = 0.67) (Δκ = +0.173; 95% CI: +0.039 to +0.321). Bland–Altman plots demonstrated narrower limits of agreement for VEA-based planning compared with the CT-based method (Supplementary Figures S1 and S2). Of note, no improvements were found using VEA when the planning was performed by expert operators in advanced cardiac imaging with no direct experience with structural heart interventions (Supplementary Scheme S1).

Reclassification analysis showed that the use of VEA resulted in an NRI of +14% (95% CI: +6-6% to +27.3%) compared with CT-based assessment. Specifically, 6 patients (14.0%) were reclassified to a better category, achieving either exact concordance with the implanted valve size or a closer estimate, while no patients were reclassified to a worse category (*p* < 0.001).

We examined whether reclassification to a more accurate prosthesis size with VEA was associated with improved clinical outcomes. The prevalence of post-procedural pacemaker implantation was numerically lower among the cases with a reclassification improvement using VEA versus the cases where no improvement was achieved using VEA, without statistical significance (risk ratio 0.28, 95% CI 0.02–4.25). No difference was registered in the prevalence of paravalvular leak (risk ratio = 1.19, 95% CI 0.78–1.80).

## 4. Discussion

The main potential of immersive VR is its ability to provide the operator with a clearer and more intuitive perception of the three-dimensional anatomy of the aortic root and surrounding structures, thereby supporting a more accurate mental reconstruction of patient-specific features (Supplementary Video S1). At the same time, since VR is based on volumetric CT data with high spatial resolution, it also offers the opportunity to explore whether anatomical diameters and other measurements relevant to preoperative planning can be reliably obtained within the virtual environment. In this study, we evaluated not only its role in enhancing anatomical understanding but also its potential use as a complementary tool for deriving procedural metrics essential to TAVI planning. In this regard, taken together, our findings indicate that VEA provides reliable and consistent anatomical measurements, supporting its potential role as a valid alternative in clinical practice. Interestingly, LVOT measurements showed a slightly lower level of agreement compared to annular assessment.

Our findings add to the growing body of evidence supporting the role of VR in structural heart interventions. Prior studies primarily emphasized the value of VR in improving procedural planning and enhancing team confidence, yet provided limited evidence on its accuracy for direct anatomical measurements [12,15,16]. In contrast, our data suggest that VR-derived measurements of the aortic annulus show excellent correlation and agreement with conventional CT analysis, thereby confirming that immersive visualization does not compromise metric precision. Notably, the slightly lower agreement observed for LVOT dimensions reflects the known variability of this parameter, underscoring the intrinsic complexity of LVOT assessment, which is influenced by its dynamic geometry, calcification burden, and methodological factors related to plane alignment and reconstruction algorithms. Although CT offers high spatial resolution and volumetric accuracy, it should be acknowledged that measurement of the LVOT remains subject to anatomical variability and methodological limitations. In fact, multiple authors documented variability in LVOT dimensions across clinical conditions and imaging modalities, including CT [18,19].

Although multidetector CT is the reference standard for pre-TAVI anatomical assessment, LVOT measurement remains challenging for several reasons. First, the LVOT has an intrinsically elliptical and dynamic geometry, which changes significantly throughout the cardiac cycle; measurements obtained at different reconstruction phases can therefore yield discrepant results. Even with ECG-gated acquisitions, subtle differences in the chosen phase (e.g., 30% vs. 75% of the cycle) may influence the calculated area and diameters. Second, the presence of extensive calcifications can often impair the accuracy of contour detection, potentially leading to either overestimation or underestimation of dimensions depending on the reconstruction algorithm. Third, operator-dependent factors such as multiplanar reformat alignment and plane orientation contribute to variability, despite the volumetric nature of CT data. Finally, inter-software differences in reconstruction algorithms and edge-detection criteria can also account for residual variability, as reported in previous comparative imaging studies [20]. These considerations highlight that, even though CT remains the most robust tool for anatomical assessment, LVOT measurement is not immune to anatomical complexity and technical limitations.

Beyond measurement reproducibility, the integration of VR into the preoperative workflow may reshape how heart teams manage patients undergoing TAVI. Immersive visualization could be systematically incorporated after conventional CT analysis, serving as an intermediate step between image acquisition and procedural decision-making. In this setting, VR would facilitate multidisciplinary discussion, particularly in anatomically complex cases, allowing interventional cardiologists and surgeons to jointly review patient-specific anatomy in an intuitive three-dimensional environment. Furthermore, VR could be coupled with procedural simulation modules, offering a safe environment to test valve sizing and positioning strategies before the actual intervention. In this regard, our data suggest that VEA benefits operators with limited experience in CT-based reconstruction by providing a more intuitive three-dimensional visualization, highlighting a potential value during training. Such integration has the potential to improve communication, streamline decision-making, and ultimately enhance patient safety and procedural outcomes. Hence, from a clinical perspective, VR offers the potential to refine pre-procedural planning by combining immersive spatial visualization with the ability to perform direct measurements. In practice, this dual capability may be particularly relevant in anatomically complex cases, such as bicuspid valves, borderline annuli, or heavily calcified LVOTs, where conventional 2D or multiplanar approaches may be limited. By fostering a clearer understanding of patient-specific anatomy, VR can support more tailored device selection, improve procedural preparedness, and potentially reduce intraoperative uncertainty. In this regard, the improved concordance observed with VEA indicates that immersive 3D visualization may allow operators to better appreciate the complex spatial relationships of the aortic root and annular anatomy, which are only partially captured by conventional two-dimensional CT reconstructions. By enhancing depth perception and facilitating real-time interaction with the anatomical model, VEA may reduce the risk of undersizing or oversizing the prosthesis, which are associated with complications such as paravalvular leak, device embolization, or annular rupture. Clinically, the higher reclassification to a more accurate size without evidence of detrimental shifts supports the potential role of VEA as a complementary tool to CT analysis, particularly in challenging anatomies. Notably, the greater accuracy achieved by the structural heart operator suggests that expertise in procedural decision-making may maximize the benefit of this technology, translating it into safer and more individualized transcatheter valve implantation. Greater accuracy in prosthesis selection can be potentially associated with improved implantation results. Similarly, a more precise prosthesis selection and implantation might improve coronary re-access, a key element in TAVI patients’ follow-up, given the progressive expansion of indications for TAVI to younger patients [4,21,22]. Moreover, immersive visualization may improve communication within Heart Teams, facilitating consensus in challenging cases and strengthening shared decision-making.

### Limitations and Future Perspectives

Despite its promise, VR-based planning still presents limitations. First, the retrospective design, the limited sample size, and the single-center nature do not allow definitive conclusions to be reached. The technology requires specific hardware and training, which may limit widespread adoption in the short term. Inter-software variability and the absence of standardized measurement protocols represent further challenges, particularly for structures such as the LVOT, whose dimensions are highly sensitive to cardiac phase selection and calcification. The valve implanted by standard clinical procedure was used as the reference comparator. This represents the real-world procedural outcome rather than a perfect benchmark. As such, the implanted valve cannot be considered a “true gold standard,” as intra-procedural factors and operator preferences may influence the final choice. The present study was not designed to analyze outcomes stratified by valve type. In fact, the distribution of both self-expanding and reflective devices is selected according to anatomical and clinical factors. Thus, no valve-specific conclusions can be drawn from the present sample. Moreover, the impact of VR on clinical outcomes remains to be validated in larger, prospective studies. Looking forward, the integration of VR with other emerging technologies—such as artificial intelligence and computational fluid dynamics—may further enhance its clinical value, paving the way for a more comprehensive, patient-specific planning approach in structural heart interventions. In this regard, the integration of multiple features and modalities to complement the anatomical characterization might further improve procedural planning. An example of complementary tool is the recently developed DASI Sim, a cloud-based simulation platform that integrates 3D reconstructions with computational flow dynamics and valve deformation modeling, aiming to predict hemodynamic outcomes. The combined use of interactive visualization and predictive modeling has a large potential to improve procedural precision and personalization. Moreover, the immersive 3D environment of VEA provides a natural framework for future AI integration, particularly for automated anatomical segmentation, annular contour detection, and valve sizing recommendation. In the near future, combining AI-assisted measurement algorithms with immersive visualization could further standardize measurements, reduce interobserver variability, and provide real-time automated prosthesis suggestions.

## 5. Conclusions

VR provides clearer and more detailed insights into aortic valve anatomy and surrounding structures. Our study is the first to demonstrate that immersive virtual reality can reliably reproduce key anatomical standard measurements of the aortic annulus and LVOT when compared with conventional CT-based analysis, while also providing a unique advantage in enhancing three-dimensional anatomical understanding. These findings support the role of VR as a complementary tool in the pre-procedural planning of TAVI, bridging intuitive visualization with reproducible metric assessment. Although further validation in larger, prospective cohorts is warranted, the integration of VR into the Heart Team workflow may contribute to more precise device selection, improved procedural preparedness, and ultimately safer and more individualized patient care.

## Figures and Tables

**Table 1:** Baseline Characteristics.

Baseline Characteristics	All N = 50
Age	80.5 (77–85.75)
Male sex	26(52)
Hypertension	43 (91)
Dyslipidemia	44 (94)
Diabetes	20 (42.5)
Prior CABG	2 (4)
CKD	12 (25.5)
Previous MI	6 (12.7)
Previous PCI	8 (17)
Prior PM	3 (6.3)
Prior Valve Surgery	1 (2.1)
Atrial Fibrillation	11 (23.4)

Data are presented as *n* (%), or as median (IQR); CABG: coronary artery bypass grafting; CKD: chronic kidney disease; MI: myocardial infarction; PCI: percutaneous coronary intervention; PM: pacemaker.

**Table 2:** Echocardiographic parameters of the study cohort.

Echocardiographic Parameters	All N = 50
EF (%)	57 (53.5–59.75)
LAVi (mL/m^2^)	47 (38.25–53.5)
Aortic V max (m/s)	4.19 (3.9–4.46)
Aortic Valve mean gradient (mmHg)	42.34 (38–51.25)
Aortic area (cm^2^)	0.7 (0.6–0.8)
sPAP (mmHg)	40 (35–45)

Data are presented as *n* (%), or as median (IQR). EF: ejection fraction. LAVi: left atrial volume index; sPAP: systolic pulmonary artery pressure.

**Table 3:** CT-derived anatomical measurements of the study cohort.

CT-Derived Anatomical Measurements	All N = 50
Mean sinotubular junction diameter	27.52 (25.5–29.55)
Mean ascending aorta diameter	33.6 (29.5–35.8)
Left coronary height	13.14 (11.54–14.72)
Right coronary height	13.7 (10.65–15.35)

Data are presented as median (IQR).

**Table 4:** Procedural characteristics of patients undergoing TAVI.

Procedural Characteristics	N = 43
Evolut FX	18 (41.8)
26 mm	6 (33.3)
29 mm	12 (66.6)
Evolut FX+	1 (2.3)
29 mm	1 (100)
Evolut Pro	1 (2.3)
26 mm	1 (100)
Evolut Pro+	4 (9.3)
26 mm	1 (25)
29 mm	3 (75)
Evolut R	2 (4.6)
34 mm	2 (100)
Navitor	6 (13.9)
25 mm	4 (66.6)
29 mm	2 (33.3)
Sapien 3 Ultra	10 (23.2)
23 mm	6 (60)
26 mm	3 (30)
29 mm	1 (10)
Sapien 3 Ultra+	1 (2.3)
26 mm	1 (100)
Pre-dilatation	10 (23)
Post-dilatation	6 (13.95)
PM post-TAVI	9 (20.1)
PCI during TAVI	6 (13.9)
Aortic regurgitation after TAVI	31 (72)
2+	9 (29.1)
Aortic mean gradient after TAVI	8.27 (6–11.25)

Data are presented as *n* (%), or as median (IQR).

## Data Availability

The raw data supporting the conclusions of this article will be made available by the authors on reasonable request.

## Supplementary Materials

The following supporting information can be downloaded at https://www.mdpi.com/article/10.3390/jcdd12120481/s1, Scheme S1: Performance of pre-procedural planning by operator expert in advanced cardiac imaging.; Figure S1: pre-procedural planning by operator expert in advanced cardiac imaging and in structural interventions.; Figure S2: Sankey diagram illustrating how patients classified as “Concordant” or “Non-concordant” with CT were reclassified when using VEA, demonstrating the shifts toward better alignment with the implanted valve size.; Video S1: example of three-dimensional pre-procedural planification.
